# 2246. A comparison between empiric carbapenem and non-carbapenem use for patients admitted with urinary tract infections and sepsis in the Dominican Republic

**DOI:** 10.1093/ofid/ofad500.1868

**Published:** 2023-11-27

**Authors:** David De Luna, Yori A Roque, Alfredo Jose Mena Lora, Danay Perez Morel, Jonas Sthir Caraballo, Francelis Vargas

**Affiliations:** Pontificia Universidad Catolica Madre y Maestra, Santiago, Santiago, Dominican Republic; Hospital Metropolitano de Santiago (HOMS), Santiago, Santiago, Dominican Republic; UIC, Chicago, Illinois; Hospital Metropolitano de Santiago, Santiago, Santiago, Dominican Republic; Pontificia Universidad Catolica Madre y Maestra, Santiago, Santiago, Dominican Republic; Pontificia Universidad Catolica Madre y Maestra, Santiago, Santiago, Dominican Republic

## Abstract

**Background:**

Septic shock causes major morbidity and mortality. Early resuscitation and empiric antimicrobial therapy is the cornerstone of therapy. Mortality can increase up to 5 times in patients with septic shock treated with an empiric regimen that does not cover the primary pathogen. Carbapenems have broad spectrum and are commonly used in sepsis. The balance of empiric use and antimicrobial stewardship can be difficult in low and middle income countries (LMIC) where multi-drug resistance (MDR) is common.

**Methods:**

This is a prospective observational study from September 1 to November 30, 2020. Patients admitted with urinary tract infection (UTI) and sepsis criteria by SOFA score were included. Patients were categorized by antibiotic into a carbapenem or non-carabpenem cohort. Data was collected prospectively and length of stay, mortality and outcome was compared between groups.

**Graphic 1.**
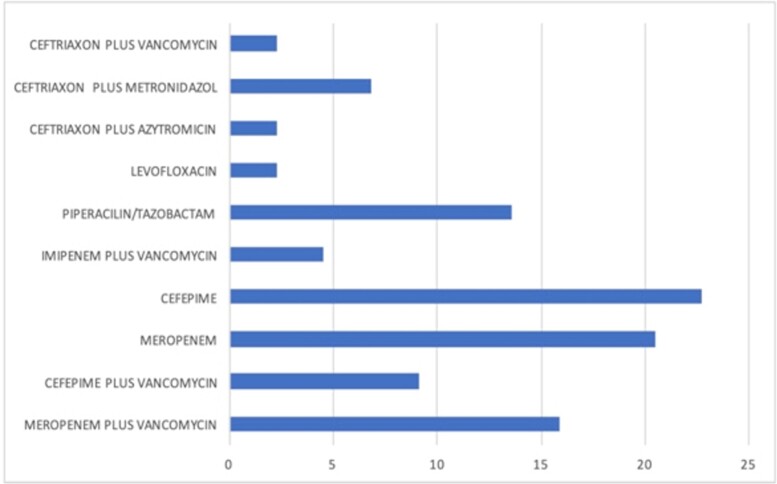
Distribution of antibiotic schemes

**Graphic 2:**
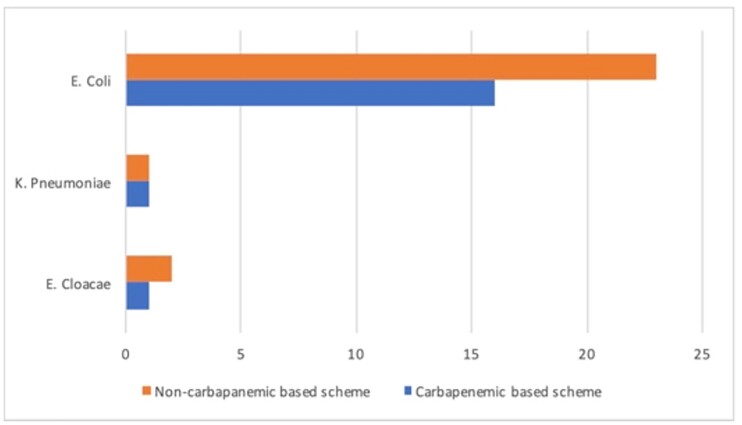
Distribution of Gram negative bacteria isolation

**Results:**

We evaluated a total of 44 patients. Most of the cases had length of stay (LOS) > 7 days (72.2%). The antibiotic most frequently used were meropenem (36.4%) and cefepime (31.8%), alone or in combination with other drug (Figure 1). Table 2 shows that there was no increased LOS or death between the two study groups; however, medical complications were more common in the non-carbapenem cohort.

**Table 2.**
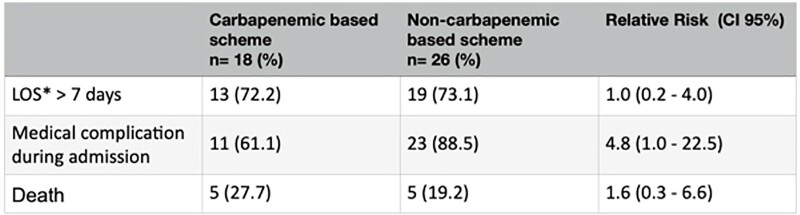
Outcome of studied population

**Conclusion:**

Overall, there were no major significant differences between the groups. We did not find an significant increased risk of death or prolonged LOS between the carbapenem and non-carbapenem cohorts, however, the appearance of complication did have an impact depending on the type of antibiotic regimen. This may offer opportunities for empiric treatment guidelines and a target for antimicrobial stewardship.

**Disclosures:**

**All Authors**: No reported disclosures

